# Molecular Mechanism of Caffeine in Preventing Bronchopulmonary Dysplasia in Premature Infants

**DOI:** 10.3389/fped.2022.902437

**Published:** 2022-06-20

**Authors:** Congliang Tian, Danni Li, Jianhua Fu

**Affiliations:** ^1^Department of Pediatrics, Shengjing Hospital of China Medical University, Shenyang, China; ^2^Department of Pediatrics, The First Affiliated Hospital of Dalian Medical University, Dalian, China

**Keywords:** bronchopulmonary dysplasia, caffeine, antioxidant, angiogenesis, anti-inflammatory, molecular mechanism

## Abstract

Bronchopulmonary dysplasia (BPD) is a chronic respiratory complication commonly seen in premature infants. Following continuous advances in neonatal intensive care diagnosis and treatment technology, an increasing number of premature babies are being treated successfully. Despite these remarkable improvements, there has been no significant decline in the incidence of BPD; in fact, its incidence has increased as more extremely preterm infants survive. Therefore, in view of the impact of BPD on the physical and mental health of children and the increased familial and social burden on these children, early prevention of BPD is emphasized. In recent decades, the clinical application of caffeine in treating primary apnea in premature infants was shown not only to stimulate the respiratory center but also to confer obvious protection to the nervous and respiratory systems. Numerous clinical cross-sectional and longitudinal studies have shown that caffeine plays a significant role in the prevention and treatment of BPD, but there is a lack of overall understanding of its potential molecular mechanisms. In this review, we summarize the possible molecular mechanisms of caffeine in the prevention or treatment of BPD, aiming to better guide its clinical application.

## Introduction

Bronchopulmonary dysplasia (BPD) is a chronic respiratory complication commonly seen in premature infants, seriously affecting their long-term quality of life (1). Despite remarkable improvements in the diagnosis and treatment of premature infants and more of these infants being successfully treated, there has been no significant decline in the incidence of BPD (2). At present, BPD lacks a comprehensive definition, let alone a specific and promoted cure (3). Preterm infants are exposed to oxidative stress at birth due to environmental changes (4). Owing to immature organ development and oxygenation therapy for respiratory instability in preterm infants, the oxidative damage is further exacerbated (5), and the infants are unable to fully adapt and mount an antioxidant defense (6). Morphological changes induced by oxidative stress lead to disruption of lung development (7) and blocked angiogenesis, accompanied by the expression of pro-inflammatory cytokines and chemokines and increased immune cell infiltration in lung tissue (8).

Over the past two decades, several therapeutic strategies for BPD have been proposed (9). Among the BPD cohorts, there are modestly beneficial treatment strategies to mitigate premature lung injury that have been described in detail in numerous reviews and surveys (10–12). These include the administration of corticosteroids (13), surfactants (12, 14), antioxidants (15), and non-invasive respiratory support (16). To date, most therapies are not effective at specifically targeting the multifactorial complex scenario observed in BPD infants, with the exception of caffeine (17). Caffeine is a respiratory stimulant that belongs to the methylxanthine class of drugs (18). In recent decades, the clinical application of caffeine to treat apnea of prematurity (AOP) in infants has been shown to not only stimulate the respiratory center but also to protect the nervous and respiratory systems (19, 20). As a non-invasive respiratory support, caffeine reduces apnea in premature infants and improves long-term lung function in children (21). A placebo-controlled trial showed that caffeine reduces the risk of BPD in preterm infants (22). Caffeine also inhibits the inflammatory response induced by hyperoxia exposure in infant rats (23).

Numerous clinical cross-sectional and longitudinal studies have shown that caffeine plays a significant role in the prevention and treatment of BPD, but there is still a lack of overall understanding of its potential molecular mechanisms. In this review, we summarize the possible molecular mechanisms of caffeine in the prevention or treatment of BPD, aiming to better guide future clinical application.

## Definition, Pathophysiology, and Impact of Bronchopulmonary Dysplasia

Bronchopulmonary dysplasia is a common chronic pulmonary disease observed in premature infants, which was first reported by Northway et al. (24). The original definition of BPD (“old” BPD) aimed to rescue premature infants with severe respiratory distress syndrome (RDS) (such as those born at around 32 weeks of gestation) with high concentrations of oxygen, high peak aspiration pressure, and other positive mechanical ventilation. These rescue approaches often resulted in pulmonary epithelial injury, smooth muscle hyperplasia, pulmonary atelectasis, and pulmonary fibrosis. With improvements in diagnostic and therapeutic modalities in neonatal intensive care, a larger number of very low-birth weight and extremely low-birth weight infants are now able to survive. Lung structure is very immature in the canalicular or saccular stage of pulmonary development, especially in very low-birth weight infants, whereby respiratory bronchioles, bronchial capillaries, and mucus glands are not fully developed, and the interstitium is not sufficiently thin to form an air-blood barrier. Additionally, the production of surfactants in pulmonary epithelial cells has not yet begun in these infants. If the fetus is delivered at this time, genetic susceptibility and adverse factors will hinder lung development, contributing to simple alveoli and abnormal development of micro-vessels, a condition referred to as “new” BPD. Excessive peak inspiratory pressure and tidal volume can lead to pulmonary barotrauma and volumetric injury and thus result in alveolar injury and a severe local inflammatory response (25, 26). A meta-analysis showed that infants of mothers with chorioamnionitis have a slightly higher risk of BPD than do infants born to control mothers (27). In addition, a higher level of reactive oxygen species (ROS) is associated with active mechanical ventilation. Antioxidant enzymes gradually mature during fetal life, and their activities gradually increase with the establishment of respiration after birth (28). Hence, even a slight increase in ROS production in premature infants can lead to lung damage. In addition, supraphysiological oxygen concentrations in experimental animals caused alveolar small airway lesions afterbirth, such as smooth muscle hyperplasia, oxidative stress, right ventricular hypertrophy, and vascular remodeling (29). Similar findings have been found in lung biopsies of newborns with neonatal RDS treated with supraphysiological oxygen concentrations and aggressive ventilation (30). In summary, the pathological process of “new” BPD begins when immature lung tissues encounter adverse stimulation, including oxidative stress, inflammatory damage, and physical damage, leading to lung development retardation and a series of clinical symptoms.

Bronchopulmonary dysplasia affects the ability of the lung to remodel during development, resulting in functional and structural abnormalities of the lung throughout childhood, adolescence, and adulthood. A survey found that 18–36 months old infants with severe BPD had a significantly lower quality of life than term infants and preterm infants without BPD (31). Several systematic reviews and meta-analyses have shown that within 2 years after birth, the readmission rate of children with BPD was as high as 50% (32–34). In addition, pulmonary vascular dysplasia in children with BPD can lead to pulmonary hypertension; a meta-analysis revealed that pulmonary hypertension occurred in 17% of children with BPD and up to 24% in children with severe BPD (35). More notably, several clinical studies have shown that BPD is an independent risk factor in neuro-dysplasia and a potential risk factor even in the absence of definite brain injury (such as intravascular hemorrhage and hypoxic-ischemic encephalopathy). For example, premature infants with BPD exhibit poorer intellectual and motor development and cognitive abilities than premature infants without BPD, presumably because lung protection strategies may also be neuroprotective (36).

## Caffeine as a Drug Therapy for Bronchopulmonary Dysplasia

In the past few years, prospective and retrospective clinical studies have been carried out on the prevention and treatment of BPD using a variety of drugs and mechanical ventilation management methods; however, there is still no unified prevention and treatment plan. In addition to the prevention of premature birth, commonly used clinical drugs include intravenous or topical corticosteroids, intravenous caffeine, inhalation of nitric oxide, stem cell therapy, vitamin A, and diuretics. Caffeine is the most commonly used drug in the NICU after antibiotics (37). In recent years, an increasing number of high-quality clinical studies have demonstrated the protective effects of caffeine on the respiratory and nervous systems of premature infants. According to a randomized controlled trial, the incidence of BPD, patent ductus arteriosus (PDA), and severe retinopathy of prematurity was lower in an early caffeine group than in a control group. Moreover, the neurological developmental outcome was better in the caffeine-treated group than in the control group (22). Previous systematic reviews and meta-analyses have shown that methylxanthine reduces the failure of extubation; thus, administration of the methylxanthine caffeine is recommended since it also reduces the failure rate of extubation (38). Caffeine also reduces the need for mechanical ventilation by improving lung function, enhancing diaphragm motor function, and increasing respiratory center sensitivity to carbon dioxide (19). Early, high doses of caffeine in the treatment of apnea in preterm infants may have a protective effect on BPD in preterm infants (39), owing to one of the most central risk factors for BPD being mechanical ventilation. A meta-analysis of 13 randomized controlled trials (40) assessed the efficacy of different maintenance doses of caffeine and showed that the high-dose group (10–20 mg/kg) had a higher rate of ventilator removal success and effective treatment, and a lower rate of simultaneous extubation failure than that of groups receiving lower doses of caffeine. Regarding the duration of caffeine therapy, several retrospective cohort studies confirmed that early (≤3 days after birth) rather than later (>3 days after birth) use of caffeine shortened the duration of mechanical ventilation (19) and reduced the morbidity and mortality of infants with BPD (41). Therefore, caffeine may reduce immature lung damage, exerting a protective effect against BPD, partly by reducing ventilator exposure (the “clinical level” effect of caffeine).

## Pharmacological Effects of Caffeine

Caffeine is a trimethylxanthine drug with three potential pharmacological activities *in vivo*, namely, adenosine receptor antagonist, phosphodiesterase inhibitor, and cellular calcium regulator (42). Adenosine is a neurotransmitter with diverse physiological functions, including the control of arousal, sleep, and cerebrovascular homeostasis. It has four known receptors: A1R, A2aR, A2bR, and A3R. Adenosine binding to its receptors leads to inhibition of inspiratory neurons, resulting in central respiratory depression. Caffeine can non-specifically block these receptors, thereby indirectly stimulating the respiratory center, increasing sensitivity to carbon dioxide, enhancing diaphragm contractility, and improving respiratory rate and tidal volume (43). Therefore, caffeine is commonly used in NICUs for the treatment of primary apnea in premature infants. With widespread clinical application, a systematic review analyzing high-quality evidence revealed that caffeine is the only drug that prevents BPD in premature infants (44).

## Mechanisms of Caffeine Prevention of Bronchopulmonary Dysplasia

The three major postnatal pathological factors of BPD comprise high-concentration oxygen inhalation, inflammatory response, and mechanical ventilation. Lung injury caused by a high concentration of oxygen and mechanical ventilation mainly includes alveolar epithelial and vascular endothelial cell necrosis or apoptosis, resulting in destruction of alveolar structure, increased vascular permeability, and recruitment of a large number of inflammatory cells leading to an inflammatory response (45). Inflammatory cells can induce local inflammation and a vascular response by producing cytokines and chemotaxis, thereby causing damage to lung tissue.

### Antioxidant Effects of Caffeine

Supplemental oxygen, a basic therapy for premature infants, is also a common treatment for BPD, but it increases oxidant stress and can result in lung injury. The fetus is in a relatively hypoxic environment *in utero*, but after birth, the atmospheric oxygen concentration is relatively high, especially for premature infants. Oxidative stress is induced by exposure to a relative or absolute high concentration of oxygen, which regulates the antioxidant capacity in the body; however, the antioxidant system is underdeveloped in premature infants. Compared with that full-term infants, the expression and activity of antioxidant enzymes in premature infants are poor. Therefore, premature infants have poor tolerance to hyperoxia, are vulnerable to ROS-mediated damage, and are more likely to suffer lung injury and development arrest (46). Ankur et al. (47) found that the accumulation of ROS in the matrix of the human body may be related to tissue damage. Numerous studies on human BPD have shown that ROS are involved in the imbalance of damage and repair during lung maturation.

**FIGURE 1:**
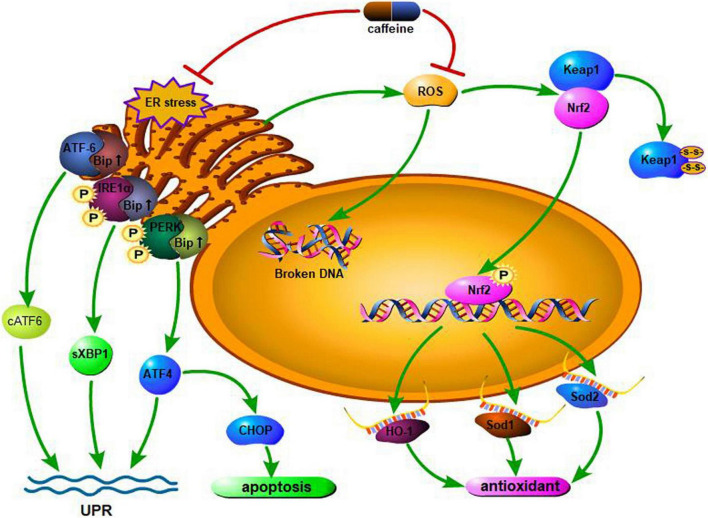
Hyperoxia increases the levels of the endoplasmic reticulum (ER) stress marker Bip and downstream effectors. On sensing the presence of ER stress, both PERK and IRE1α are phosphorylated, activating downstream effectors, while ATF-6 is cleaved. Through the above reactions, the body initiates the unfolded protein response (UPR), alleviating oxidative stress damage. If the stress persists, the UPR becomes ineffective. Cells will undergo CHOP-mediated apoptosis if the UPR fails to mitigate the ER stress. Caffeine can attenuate all of the responses. In addition, when excessive ROS are generated upon exposure to hyperoxia, caffeine administration can prevent and/or reduce hyperoxia-induced ROS generation and any resultant DNA damage. In response to oxidative stress, the expression of antioxidant genes is regulated by the Nrf2/Keap1 pathway. Expression of Nrf2, a cytoprotective mediator that regulates the expression of genes encoding antioxidant, anti-inflammatory, and detoxifying proteins is induced by hyperoxia, while expression of Keap1 is repressed. Caffeine inversely regulates RNA expression. The expression of Sod1, Sod2, and HO-1 are regulated by Nrf2. ROS, reactive oxygen species; IRE1α, inositol-requiring enzyme 1α; cATF6, cleaved form of activating transcription factor 6; PERK, PKR-like ER kinase; Bip, binding immunoglobulin protein; sXBP1, spliced X-box binding protein 1; CHOP, CCAAT/enhancer binding protein homologous protein; ATF4, activating transcription factor 4; Nrf2, NFE2-related factor 2; Keap1, Kelch-like ECH-associated protein 1; HO-1, heme oxygenase-1; Sod1, superoxide dismutase 1; Sod2, superoxide dismutase 2.

Hyperoxia produces a large amount of ROS, which can lead to DNA strand breaks that mainly affect cell proliferation and type II alveolar epithelial cells. In addition, ROS-induced lipid peroxidation can alter cell membrane permeability and fluidity, thereby disrupting cell integrity and promoting cell apoptosis. The Nrf2/Keap1 pathway mediates the regulation of oxidative stress through the controlled expression of antioxidant genes; Nrf2 is a cellular protective transcription factor involved in regulating the expression of genes encoding antioxidant, anti-inflammatory, and detoxifying proteins (48). Endesfelder et al. (49) found that in a hyperoxia-based murine BPD model, exposure to high concentrations of oxygen induced oxidative stress and lipid peroxidation, increased Nrf2 levels, and decreased Keap1 levels; this response could be alleviated by caffeine as an effective antioxidant medium. Moreover, caffeine can significantly enhance superoxide dismutase (Sod) gene expression, eliminate the lack of oxygen-induced Sod, and reduce oxidative damage to DNA, thus altering the morphological changes in alveoli and pulmonary vessels. Two responses, namely, endoplasmic reticulum (ER) stress and impaired mitochondrial function, can lead to hyperoxia-induced impaired lung development in rodents. The ER mediates protein synthesis, folding, and modification in the eukaryotic cell; it is sensitive to oxidative stress and responds with the help of the unfolded protein response. At the early stage of oxidative stress, ER-mitochondrial coupling is increased, thus promoting mitochondrial function and cellular adaptation to stress (50). However, chronic ER stress leads to ROS formation, releasion, and amplification of the inflammatory response. This is followed by apoptosis through mitochondria-dependent and independent pathways, leading to impaired angiogenesis and vasodilation. Angiogenesis plays a crucial role in lung development after birth (51); impaired angiogenesis is involved in the progression of BPD. Caffeine is a non-selective phosphodiesterase inhibitor that acts as a chemical chaperone, reducing ER stress (52) and potentially helping to protect against oxidative stress damage in the lungs. Teng et al. (53) found that hyperoxia exposure increased the expression of Bip, PERK, IRE1, sXBP1, cATF6, and CHOP during the cystic and alveolar stages of lung development, which enhanced oxidative stress and ER stress leading to lung damage. Caffeine can reverse this oxidative damage, reduce apoptosis, and promote angiogenesis and alveolar development. Figure 1 describes the molecular mechanism of the antioxidant effects of caffeine.

### Caffeine Promotes Angiogenesis and Improves Tissue Remodeling

**FIGURE 2:**
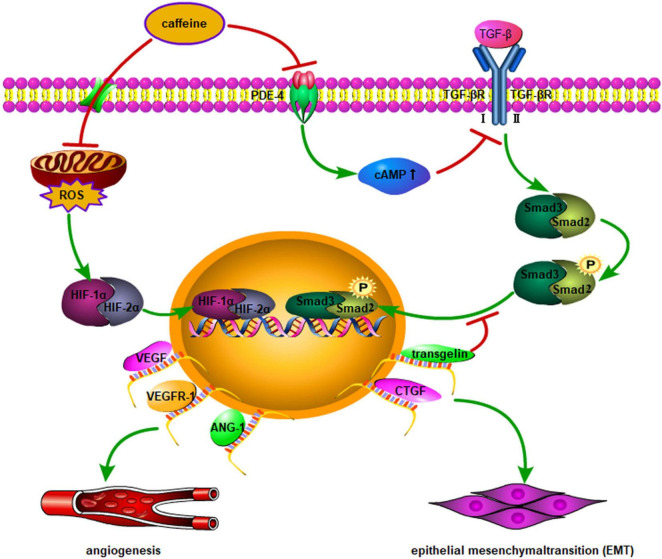
Multiple genes are involved in angiogenesis and vascular remodeling though nuclear transcription activated by HIF-1α/2α. In addition to vascular remodeling, a higher expression level of angiopoietin-1, VEGF, and VEGFR1 may normalize lung histology in hyperoxia-induced lung injury after caffeine administration. Moreover, the effect of caffeine on Smad signaling appears to be mediated by inhibition of PDE-4, leading to an increase in intracellular cAMP, which is a possible regulator of TGF-β-mediated Smad phosphorylation and nuclear translocation. EMT activation is triggered by upregulation of CTGF and transgelin through the above-mentioned pathways. ROS, reactive oxygen species; TGF-β, transforming growth factor-β; TGF-βR, TGF-β receptor; PDE-4, phosphodiesterase-4; CTGF, connective tissue growth factor; cAMP, cyclic adenosine monophosphate; VEGF, vascular endothelial growth factor; VEGFR-1, VEGF receptor-1; EMT, epithelial mesenchymal transition; ANG-1, angiopoietin-1; HIF-1α, hypoxia-inducible factor-1α; HIF-2α, hypoxia-inducible factor-2α.

The main manifestation of “new” BPD is the arrest of lung developmental imbalance of damage and repair due to prenatal or postnatal factors, leading to alveolar simplification and pulmonary microvascular developmental disorders. Angiogenesis is essential for the formation of alveolarization and differentiation during normal lung development (54). Recently, numerous studies have shown that vascular endothelial growth factor (VEGF) plays a key role in the pathogenesis of BPD (54, 55). Hypoxia-inducible factor-1 (HIF-1) regulates the expression of genes encoding vascular development, among which *VEGF* and *ANGPT1* are important genes. The expression of HIF-1 is closely related to oxygen concentration in the body. Exposure to high concentrations of oxygen, or even air, in developing lungs rapidly induces degradation of HIF, leading to downregulation of VEGF expression. Dumpa et al. (56) found that HIF-2α, VEGF receptor-1 (VEGFR1), and angiopoietin (ANGPT1) were highly expressed in a hyperoxia group treated with caffeine. The observed increase in radial alveolar count and vascular surface area, and decrease in mean linear intercept and smooth muscle thickness of pulmonary arterioles, suggest that caffeine can alleviate hyperoxic lung injury and promote pulmonary vascular development. In particular, caffeine exerts a protective effect on vascular remodeling in male mice. The transforming growth factor (TGF) family of proteins plays a key role in lung development; the TGF-β signaling pathway is pathogenic in patients with BPD. Rath et al. (57) found that caffeine downregulated the expression of TGF-βR1, TGF-βR3, and Smad2 *in vitro*. *In vivo* experiments revealed increased expression of TGF-βR1, TGF-βR2, and the second messengers Smad2 and Smad3 in a hyperoxia-induced BPD murine model. Caffeine can reverse connective tissue growth factor (CTGF) expression, which is linked to alveolar remodeling; however, it has no protective effect on the morphology of lungs damaged by hyperoxia. Moreover, in mice, lungs mature slowly after birth; thus, this conclusion may be related to the experimental oxygen concentration application, administration time, and excessive dosing. Mice are more susceptible to oxidative stress than rats, and excessive use of caffeine may lead to an increased inflammatory response. Fehrholz et al. (58) found that as a non-specific phosphodiesterase inhibitor, caffeine could regulate the expression of CTGF and transgelin (a cytoskeletal binding and stabilizing protein) through the TGF-β/Smad pathway, and change the remodeling of airway epithelial cells, thereby achieving a protective effect against BPD on lung damage. Figure 2 describes the molecular mechanism of caffeine promotion of angiogenesis and improvement in tissue remodeling.

### Anti-inflammatory Effects of Caffeine

**FIGURE 3:**
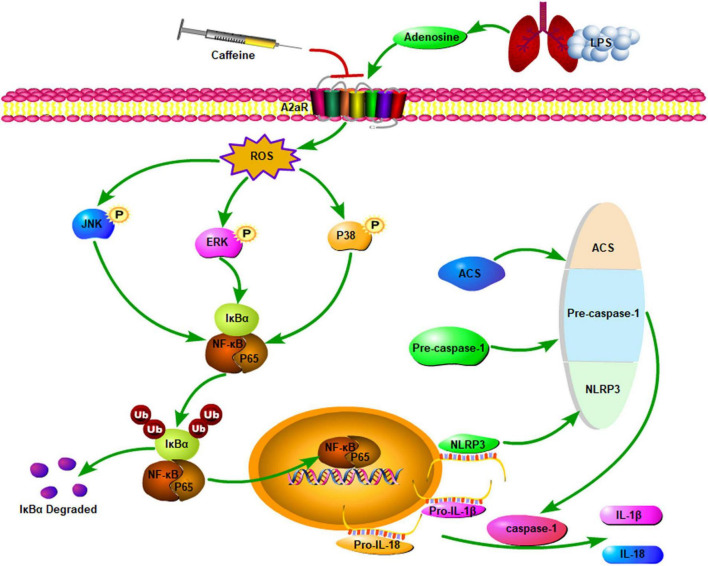
Caffeine antagonism of A2aR reduces ROS production and subsequent phosphorylation levels of MAPK and NF-κB pathway members, thus inhibiting NF-κB translocation to the nucleus and reducing NLRP3 expression, ASC speck formation, and caspase-1 cleavage. This ultimately leads to a reduction in the secretion of IL-18 and IL-1β. A2aR, adenosine 2a receptor; LPS, lipopolysaccharide; ROS, reactive oxygen species; ERK, extracellular regulated protein kinases; JNK, c-Jun N-terminal kinase; NLRP3, nucleotide-binding domain and leucine-rich repeat protein 3; ACS, apoptosis associated speck-like protein containing a CARD; NF-κB, nuclear factor kappa-light-chain-enhancer of activated B cells; IκB, inhibitor of κB; IL, interleukin.

The pathogenesis of BPD is actually multifactorial and mainly characterized by alveolar and vascular growth arrest as mentioned above, which is associated with inflammation. Prenatal inflammatory exposure of the fetus is mainly due to chorioamnionitis, including clinical and histological chorioamnionitis, according to its pathogenesis. Chorioamnionitis affects lung development of the fetus, which may be harmful or beneficial to premature infants. Histological chorioamnionitis reduces the risk of RDS, possibly owing to increased production of pulmonary surfactant. However, infants who are exposed to chorioamnionitis require longer respiratory support, resulting in a higher rate of BPD and neonatal persistent pulmonary hypertension, despite a lower risk of RDS (59). Although the incidence of BPD following exposure to chorioamnionitis is complicated by low-weight birth and various postnatal complications, the incidence of BPD in infants exposed to chorioamnionitis for whom long-term mechanical ventilation is avoided is lower than in neonates treated with chronic mechanical ventilation (60). It is uncertain whether prenatal chorioamnionitis exposure and postnatal lung inflammation, which is induced by being on a ventilator, are important factors in the pathogenesis of BPD. Intraamniotic injection of lipopolysaccharide (LPS) to induce chorioamnionitis in fetal lambs resulted in abnormal lung development following exposure to inflammation; this was consistent with observed alveolar hypoplasia, impaired alveolar surfactant secretion, and impaired pulmonary vascular development and function in infants who died from BPD (61). Mechanical ventilation can increase the infiltration of neutrophils and macrophages into the alveoli. These inflammatory cells produce cytokines (IL-1β, IL-6, and IL-8) that disrupt lung development, and the levels of these pro-inflammatory cytokines remain elevated (62). In premature lambs, mechanical ventilation for 2 h caused lung inflammation, and prolonged ventilation time (3–4 weeks) increased neutrophil and macrophage infiltration in the lungs. This resulted in uneven lung inflation and abnormal development of the pulmonary vasculature, which was similar to the pathological changes observed in the lungs of infants who died of BPD (63). Recent evidence demonstrated that caffeine inhibits the secretion of pro-inflammatory cytokines (particularly IL-1β), reducing hyperoxia and LPS-induced inflammation in rodent models (64). Weichelt et al. (23) exposed 6-day-old pups to 80% hyper-oxygen for 24 h and observed the infiltration of neutrophils and macrophages in the lungs; after caffeine intervention, the expression of neutrophil chemoattractant-1, macrophage inflammatory protein-2, tumor necrosis factor (TNF)-α, and IL-6 was upregulated, while the expression of inflammatory cells and cytokines was downregulated to baseline levels. This suggests that the protective effect of caffeine on newborn lungs is mediated, at least in part, by reducing lung inflammation. Activation of the NLRP3 inflammasome is associated with the pathogenesis of acute lung injury. Zhao et al. (65) found that NLRP3 expression was significantly decreased by caffeine and the activity of ASC spot protein and caspase-1 cleavage *in vitro*, thereby reducing IL-1β and IL-18 secretion by THP-1 macrophages. Furthermore, the levels of MAPK and NF-κB pathway phosphorylation members were significantly decreased, which further inhibited NF-κB migration in THP-1 macrophages. The expression of caspase-1 was significantly reduced through silencing of the adenosine A2a receptor (A2aR) in THP-1 macrophages, thereby reducing ROS production. In summary, caffeine reduces ROS production by antagonizing A2aR in LPS-induced THP-1 macrophages, which modulates MAPK/NF-κB signaling and inhibits NLRP3 inflammasome activation. Chen et al. (66) found that caffeine significantly reduced oxidative stress, promoted alveolar development, and alleviated apoptosis caused by inflammatory infiltration and lung injury by decreasing A2aR protein expression. In turn, this inhibited the NLRP3 inflammasome and the NF-κB pathway in hyperoxic lung injury, both *in vitro* and *in vivo*, thereby protecting lung tissue from BPD-related damage. Endesfelder et al. (17) found that the infiltration of macrophages, neutrophils, and other inflammatory cells in the lungs was reduced in the caffeine-treated group in a hyperoxic BPD model compared with that in the control group. Furthermore, caffeine downregulated the expression of TNFα, IL-1α, and IL-1β through the NF-κB pathway, which reduced apoptosis of lung epithelial cells; the caffeine target was mainly related to downregulated expression of A2aR. Figure 3 describes the molecular mechanism of caffeine in anti-inflammatory effects.

### Other Effects of Caffeine

Caffeine not only reduces the incidence of BPD at the molecular level but also alleviates BPD by improving lung function. An observational retrospective clinical study of the effect of caffeine treatment on lung function, for example, showed that early caffeine treatment increased lung capacity in preterm infants with or without BPD (67). In addition, a single-center randomized controlled clinical study showed an increased forced respiratory flow rate during childhood in premature infants treated with caffeine early, which might be related to the promotion of healthy lung development by early caffeine use (68). Caffeine also improves lung compliance. Fehrholz et al. (69) demonstrated that caffeine enhanced the effects of glucocorticoids in promoting alveolar surfactant production, maturation, and release; Nagatomo et al. (70) reached a similar conclusion. The potential mechanism involves caffeine protecting lung tissue by improving lung compliance, thereby reducing barotrauma and shear injury during mechanical ventilation and reducing ventilator duration. Patent ductus arteriosus (PDA) is also an important risk factor of BPD. Caffeine may improve the prognosis of BPD by antagonizing the effect of prostaglandins on ductus arteriosus closure. However, a study of ductus arteriosus in preterm fetal sheep found no direct relationship between the therapeutic concentration of caffeine and ductus tension (71). Moreover, a small clinical observational study found that intravenous caffeine administration increased the left-to-right shunt in PDA (72). Therefore, whether caffeine can improve the prognosis of BPD by reducing the incidence of PDA requires additional study. Furthermore, an animal study showed that caffeine exerted a diuretic effect by antagonizing adenosine A1 receptors in renal tubules (73). The diuretic effect of caffeine may improve the prognosis of BPD by reducing the occurrence of pulmonary edema. However, to date, there is no direct clinical evidence that caffeine prevents BPD through diuretic effects.

## An Urgent Problem to Be Solved

Bronchopulmonary dysplasia is common in premature infants, especially those younger than 28 weeks of gestational age. The pathological changes are due to prenatal or postnatal harmful factors acting on immature lungs, resulting in stagnation of lung development. BPD is the result of multiple factors; thus, prevention and treatment require a comprehensive management process. Although an increasing number of studies have investigated the pathogenesis of BPD in recent years, the complexity of the pathogenesis has impeded a full understanding of the mechanisms leading to lung injury. This explains why there are so many clinical treatment methods but no standard therapeutic strategy for BPD. For nearly half a century, caffeine has been widely recognized as a treatment for primary apnea in premature infants, and it has been found to prevent BPD in premature infants. Although caffeine is widely used in clinical practice, there are still some shortcomings. In particular, Dayanim et al. (74) showed that caffeine treatment exacerbated hyperoxic lung injury in neonatal rats by increasing alveolar cell apoptosis. In addition, the timing, dosage, and side effects of caffeine use need to be further examined.

## Author Contributions

CT prepared the preliminary draft of the review. DL and JF critically revised the manuscript for intellectual content. All authors contributed to the article and approved the submitted version.

## Conflict of Interest

The authors declare that the research was conducted in the absence of any commercial or financial relationships that could be construed as a potential conflict of interest.

## Publisher’s Note

All claims expressed in this article are solely those of the authors and do not necessarily represent those of their affiliated organizations, or those of the publisher, the editors and the reviewers. Any product that may be evaluated in this article, or claim that may be made by its manufacturer, is not guaranteed or endorsed by the publisher.
